# Day‐by‐day blood pressure variability in hospitalized patients with COVID‐19

**DOI:** 10.1111/jch.14338

**Published:** 2021-07-31

**Authors:** Fei‐Ka Li, De‐Wei An, Qian‐Hui Guo, Yi‐Qing Zhang, Jia‐Ye Qian, Wei‐Guo Hu, Yan Li, Ji‐Guang Wang

**Affiliations:** ^1^ Department of Geriatrics Ruijin Hospital Shanghai Jiaotong University School of Medicine Shanghai China; ^2^ The Shanghai Institute of Hypertension Ruijin Hospital Shanghai Jiaotong University School of Medicine Shanghai China; ^3^ Department of General Surgery Ruijin Hospital Shanghai Jiaotong University School of Medicine Shanghai China

**Keywords:** blood pressure variability, COVID‐19, inpatient, mortality

## Abstract

In a retrospective analysis, the authors investigated day‐by‐day blood pressure variability (BPV) and its association with clinical outcomes (critical vs. severe and discharged) in hospitalized patients with COVID‐19. The study participants were hospitalized in Tongji Hospital, Guanggu Branch, Wuhan, China, between February 1 and April 1, 2020. BPV was assessed as standard derivation (SD), coefficient of variation (CV), and variability independent of mean (VIM). The 79 participants included 60 (75.9%) severe patients discharged from the hospital after up to 47 days of hospitalization, and 19 (24.1%) critically ill patients transferred to other hospitals for further treatment (*n* = 13), admitted to ICU (*n* = 3) or died (*n*=3). Despite similar use of antihypertensive medication (47.4% vs. 41.7%) and mean levels of systolic/diastolic blood pressure (131.3/75.2 vs. 125.4/77.3 mmHg), critically ill patients, compared with severe and discharged patients, had a significantly (*p* ≤ .04) greater variability of systolic (SD 14.92 vs. 10.84 mmHg, CV 11.39% vs. 8.56%, and VIM 15.15 vs. 10.75 units) and diastolic blood pressure (SD 9.38 vs. 7.50 mmHg, CV 12.66% vs. 9.80%, and VIM 9.33 vs. 7.50 units). After adjustment for confounding factors, the odds ratios for critical versus severe and discharged patients for systolic BPV were 3.41 (95% confidence interval [CI] 1.20‐9.66, *p *= .02), 4.09 (95% CI 1.14‐14.67, *p *= .03), and 2.81 (95% CI 1.12‐7.05, *p *= .03) for each 5‐mmHg increment in SD, 5% increment in CV, and 5‐unit increment in VIM, respectively. Similar trends were observed for diastolic BPV indices (*p* ≤ .08). In conclusion, in patients with COVID‐19, BPV was greater and associated with worse clinical outcomes.

## 1 INTRODUCTION

The SARS‐COV‐2 (COVID‐19) pandemic is still ongoing worldwide. Early studies repeatedly showed that, in the presence of hypertension, the disease of COVID‐19 was more severe and associated with a higher fatality.^1^ Because angiotensin converting enzyme (ACE) 2 behaves as a mediator of the COVID‐19 infection, early views focused on the use of ACE inhibitors or angiotensin receptor blockers that may potentially increase the expression of ACE2 in several tissues, though not necessarily the lungs.^2^ Most studies on this topic did not show any significant association.^3^, ^4^

The results of subsequent studies suggested that hypertension itself and its target organ damage and complications might be the cause of high risk and fatality.^5^ We hypothesize that blood pressure fluctuates on critical conditions, such as COVID‐19, and increases the severity and fatality of the disease. There is some evidence on the negative health consequences of the day‐by‐day blood pressure variability (BPV) during hospitalization on critical disease conditions.^6^ In the present study, we investigated the day‐by‐day clinic BPV and its association with clinical outcomes in hospitalized patients with COVID‐19.

## 2 METHODS

The data that support the findings of this study are available from the corresponding author upon reasonable request.

### 2.1 Study population

The study participants were hospitalized patients with COVID‐19 in Tongji Hospital, Guanggu Branch, Wuhan, China, between February 1 and April 1, 2020, because of severe or critical disease conditions. The whole clinical team was from Ruijin Hospital, Shanghai Jiaotong University school of Medicine, Shanghai, China. All patients were ≥18 years of age and had COVID‐19 diagnosed according to the Diagnosis and Treatment Protocol for Novel Coronavirus Pneumonia (Preliminary version 6) of the Chinese National Health Commission.^7^ The present retrospective analysis was approved by the Ethics Committee of Ruijin Hospital and conducted in accordance with the declaration of Helsinki with patients’ informed consent exempted.

During the whole study period, a total of 90 patients were hospitalized. In this retrospective analysis, we attempted to include as many patients as possible. We did not apply any exclusion criteria except for the minimum required number of blood pressure readings for the computation of BPV indices. We had to exclude 11 patients with less than three blood pressure readings obtained during the whole hospitalization period. Thus, the present analysis included 79 patients.

### 2.2 Blood pressure and other clinical, anthropometric and biochemical measurements

Blood pressure was measured in the morning usually at 06:00‐10:00 in the supine position by a doctor or nurse using a validated electronic blood pressure monitor (Omron J30, an equivalence of HEM‐7130 in the China market, Omron Healthcare, Kyoto, Japan)^8^ before breakfast and intake of antihypertensive drugs. One reading was obtained on each occasion. Mean arterial pressure was calculated as the sum of a third of systolic blood pressure plus two thirds of diastolic blood pressure.

Information on other clinical, anthropometric, and biochemical measurements was also collected from the medical records. Body height and body weight were self‐reported. Body mass index was calculated as the body weight in kilograms divided by the body height in meters squared. Estimated glomerular filtration rate (eGFR) was calculated by the use of the CKD‐EPI formula.^9^

### 2.3 Symptoms, treatment, and clinical outcomes of COVID‐19

Information on the diagnosis, symptoms, disease severity, treatment, and clinical outcomes of COVID‐19 was collected from medical records. Oxygen saturation was measured with pulse oximetry. Hypoxia was defined as an oxygen saturation of ≤93%.^7^ Adverse clinical outcomes included transfer to other hospitals for further treatment, ICU admission or death due to COVID‐19 exacerbation. Patients were discharged at doctor's discretion when the discharge requirements were met, including (1) normothermia, (2) significant recovery of respiratory symptoms, (3) significant improvement of acute diffusive lesion of the lung identified by imaging, and (4) two consecutive negative results of COVID‐19 nucleic acid testing within an interval of at least one day. Patients were classified as “severe” if discharged and “critical” if transferred to other hospitals for further treatment, admitted to ICU, or died.

### 2.4 Statistical analysis

Statistical analyses were performed in R (Version 4.0.5, R Core Team, Vienna, Austria). Continuous analyses were performed using the Student *t* test or Mann–Whitney *U* test depending on normality, and categorical analyses using the Fisher's exact test. BPV was computed as standard deviation (SD), coefficient of variation (CV), and variability independent of mean (VIM). Odds ratios (OR, 95% confidence interval [CI]) were computed using adjusted logistic regression models, which included age, sex, and other covariables showing a significant between‐group difference in the univariate analysis. A two‐tailed *p* value of < .05 was considered statistically significant.

## 3 RESULTS

### 3.1 Patients’ demographics, disease history, and blood chemistry at admission

The 79 study participants included 60 (75.9%) severe patients discharged from the hospital after up to 47 days of hospitalization, and 19 (24.1%) critically ill patients transferred to other hospitals for further treatment (*n* = 13, 16.5%), admitted to ICU (*n* = 3, 3.8%), or died (*n* = 3, 3.8%). The duration of blood pressure measurement was 3–47 days (median 18 days). During follow‐up, blood pressure was measured every day in 15 (19.0%), at least every other day in 41 (51.9%), and even less frequently in 23 (29.1%) patients.

Table 1 shows the patients’ demographics, self‐reported disease history, and blood chemistry at admission by clinical outcome. Critically ill patients, compared with severe patients, were older (66.6 vs. 59.1 years, *p *= .02), and had lower serum total cholesterol (3.49 vs. 4.09 mmol/L, *p *= .03). They had similar sex distributions, body mass index, current smoking, self‐reported disease history, and blood chemistry measurements other than serum total cholesterol at admission (*p* ≥ .06).

**TABLE 1:** Patients’ demographics, self‐reported disease history and blood chemistry at admission by clinical outcome

Variable	Critical (*n* = 19)	Severe (*n* = 60)	*p*
Demographics			
Age, years	66.6 ± 11.2	59.1 ± 13.6	.02
Male sex, *n* (%)	7 (36.8)	30 (50.0)	.43
Body mass index (kg/m^2^)	23.3 ± 2.8	23.5 ± 3.3	.80
Current smoking	1 (5.3)	3 (5.0)	.99
Self‐reported disease history, n (%)			
Hypertension	6 (31.6)	23 (38.3)	.79
Diabetes mellitus	2 (10.5)	7 (11.7)	.99
Dyslipidemia	2 (10.5)	11 (18.3)	.72
Cardiovascular disease	0 (0.0)	3 (5.0)	.99
Blood chemistry at admission			
Fasting plasma glucose (mmol/L)	7.49 ± 3.25	6.64 ± 4.46	.38
Serum total cholesterol (mmol/L)	3.49 ± 1.03	4.09 ± 0.98	.03
Serum triglycerides (mmol/L	1.33 (1.08–1.80)	1.42 (1.13–2.00)	.45
Serum HDL cholesterol (mmol/L	0.87 ± 0.26	1.05 ± 0.42	.06
Serum LDL cholesterol (mmol/L	2.25 ± 0.93	2.68 ± 0.73	.13
Serum uric acid (mmol/L)	265.6 ± 140.1	261.8 ± 83.2	.91
Serum creatinine (mmol/L)	66 (56 ‐ 89)	71 (56 ‐ 84)	.74
eGFR (ml/min/1.73m^2^)	77.5 ± 27.6	90.4 ± 20.5	.11

Values are mean ± SD, median (interquartile range) or number of patients (% of column total).

*Abbreviations*: eGFR, estimated glomerular filtration rate; HDL, high density lipoprotein; LDL, low density lipoprotein.

### 3.2 Major clinical characteristics during hospitalization

Table 2 shows the major clinical characteristics during hospitalization by clinical outcome. Critically ill and severe patients were similar in the use of various antihypertensive drugs, mean levels of systolic and diastolic blood pressure and heart rate during hospitalization, and most of the COVID‐19 symptoms and treatments (*p* ≥ .10). However, critically ill patients had a significantly lower minimum oxygen saturation (80.4% vs. 93.0%, *p *= .02), higher proportion of fever (100% vs. 78.3%, *p *= .03) and hypoxia (84.2% vs. 45.0%, *p *= .003), and higher use of glucocorticoids (57.9% vs. 13.3%, *p *< .001).

**TABLE 2:** Major clinical characteristics during hospitalization by clinical outcome

Variable	Critical (*n* = 19)	Severe (*n* = 60)	*p*
Use of antihypertensive drugs, *n* (%)			
Any	9 (47.4)	25 (41.7)	.79
Angiotensin‐converting enzyme inhibitors	2 (10.5)	2 (3.3)	.24
Angiotensin receptor blockers	2 (10.5)	4 (6.7)	.63
Other classes of drugs	9 (47.4)	24 (40.0)	.60
Blood pressure and heart rate			
Median number of readings (range)	15 (3–46)	13 (3–40)	.53
Systolic blood pressure			
Mean (mmHg)	131.3 ± 15.9	125.4 ± 12.4	.15
SD (mmHg)	14.92 ± 5.60	10.84 ± 4.23	.007
CV (%)	11.39 ± 3.69	8.56 ± 3.00	.005
VIM (units)	15.15 ± 5.21	10.75 ± 3.69	.002
Diastolic blood pressure			
Mean (mmHg)	75.2 ± 7.8	77.3 ± 7.9	.33
SD (mmHg)	9.38 ± 3.36	7.50 ± 2.82	.04
CV (%)	12.66 ± 4.78	9.80 ± 3.81	.03
VIM (units)	9.33 ± 3.35	7.50 ± 2.84	.04
Heart rate (beats/minute)	80.7 ± 12.4	78.5 ± 7.1	.47
Minimum oxygen saturation (%)	80.4 ± 21.9	93.0 ± 2.6	.02
COVID‐19 symptoms, n (%)			
Fever	19 (100.0)	47 (78.3)	.03
Cough	17 (89.5)	47 (78.3)	.50
Sputum	11 (57.9)	29 (48.3)	.60
Dyspnea	10 (52.6)	18 (30.0)	.10
Hypoxia	16 (84.2)	27 (45.0)	.003
COVID‐19 treatment, n (%)			
Antibiotics	15 (78.9)	51 (85.0)	.50
Antivirals	18 (94.7)	56 (93.3)	.99
Glucocorticoids	11 (57.9)	8 (13.3)	<.001

Values are mean ± standard deviation (SD) or number of patients (% of column total), unless indicated otherwise.

*Abbreviations*: COVID‐19, coronavirus disease 2019; CV, coefficient of variation.

**FIGURE 1:**
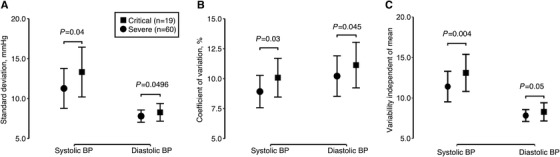
Day‐by‐day clinic blood pressure variability of standard deviation (A), coefficient of variation (B), and variability independent of mean (C) by clinical outcome. Symbols represent mean values in severe (dots) and critically ill (squares) patients, adjusted for age, sex, and the mean level of systolic and diastolic blood pressure and pulse rate, and presence of hypoxia during treatment. *p* values for the comparison between severe and critically ill patients are given

Despite similar use of antihypertensive medication (47.4% vs. 41.7%) and mean levels of systolic/diastolic blood pressure during treatment (131.3/75.2 vs. 125.4/77.3 mmHg), critically ill patients, compared with severe patients, had a significantly (*p* ≤ .04) greater systolic BPV for SD (14.92 vs. 10.84 mmHg), CV (11.39% vs. 8.56%), and VIM (15.15 vs. 10.75) on the basis of 3 to 46 blood pressure readings (Figure 1). Similar trends were observed for diastolic BPV.

### 3.3 BPV and clinical outcomes

In a logistic regression model, we included age and sex and the confounding factors with a significant between‐group difference identified in Tables 1 and 2. After multivariable adjustment, systolic BPV indices were significantly (*p* ≤ .03) associated with worse clinical outcomes. The corresponding ORs were 3.41 (95% CI 1.20–9.66, *p *= .02), 4.09 (95% CI 1.14–14.67, *p *= .02), and 2.81 (95% CI 1.12–7.05, *p *= .03) for each 5‐mmHg increment in SD, 5% increment in CV, and 5‐unit increment in VIM, respectively. The corresponding adjusted odds ratios for diastolic BPV indices were 3.51 (95% CI 0.89–13.82, *p *= .07), 2.69 (95% CI 0.97–7.52, *p *= .06), and 3.38 (95% CI 0.87–13.17, *p *= .08), respectively (**Table** **3**). Further adjustment for the duration of BP measurement slightly weakened the associations but did not materially alter the results (data not shown).

**TABLE 3:** Association of blood pressure variability and other factors with the risk of adverse clinical outcomes

Variable	Odds ratio (95% confidence interval)	*p*
Systolic blood pressure		
SD (+5 mmHg)	3.41 (1.20–9.66)	.02
CV (+5%)	4.09 (1.14–14.67)	.03
VIM (+5 units)	2.81 (1.12–7.05)	.03
Diastolic blood pressure		
SD (+5 mmHg)	3.51 (0.89–13.82)	.07
CV (+5%)	2.69 (0.97–7.52)	.06
VIM (+5 units)	3.38 (0.87–13.17)	.08

Adjusted variables consist of age, sex, and all significant variables presented in Table 1, 2, including fever, hypoxia, use of glucocorticoid, and serum total cholesterol.

## 4 DISCUSSION

The key finding of the present study was that the critically ill patients had increased systolic and diastolic BPV, which was associated with worse clinical outcomes. Regardless of whether the assessed BPV is a risk indicator or factor, it may serve as an important biological marker for clinical outcomes of COVID‐19.

Numerous previous studies had investigated the association of hypertension and various antihypertensive drugs with clinical outcomes in patients with COVID‐19. There is consensus that patients with hypertension have worse clinical outcomes,^10^ whereas the use of antihypertensive drugs have neutral^11^ or even protective effects.^12^ However, there is very limited evidence on the association between BPV and clinical outcomes in COVID‐19.

Why and how the increased BPV was associated with the disease severity and clinical outcomes of COVID‐19 is incompletely understood. One of the possible explanations might be that the illness of COIVD‐19 caused both increased BPV and worse clinical outcomes. COVID‐19 features extrapulmonary organ damage including the heart and kidneys.^13^ Several mechanisms such as systemic inflammation, disrupted cardiopulmonary coupling, cardiac insufficiency, and decreased kidney perfusion, may compromise BP regulation and lead to increased BPV before the final circulatory failure. Indeed, in a recent study in patients with COVID‐19 and hypertension, Nam and coworkers reported that higher mean arterial pressure variability was associated with a higher risk of mortality as outcome and with older age, higher C reactive protein concentration, and markers of cardiac and renal injury as the related risk factors.^14^ However, it is also possible that the disrupted blood pressure regulation or the too high or too low blood pressure as indicated by increased BPV directly caused worse clinical outcomes, such as shock.

The study should be interpreted within the context of its limitations. The sample size was rather small and hence inadequately powered, but nevertheless allowed detection of significant associations between BPV and clinical outcomes. In addition, for the sake of minimum intervention, only one blood pressure reading was obtained on each measurement occasion. However, blood pressure was measured for the whole study period.

In conclusion, in hospitalized patients with COVID‐19, day‐by‐day clinic BPV was higher and associated with worse clinical outcomes. The finding pinpoints the importance of blood pressure monitoring and further investigation for the underlying causes of increased BPV in patients with COVID‐19.

## CONFLICT OF INTEREST

Dr JG Wang reports having received lecture and consulting fees from Novartis, Omron, Servier and Takeda. The other authors declare no conflict of interest.

## AUTHOR CONTRIBUTIONS

Yan Li contributed to the conception and design of the study. Fei‐Ka Li acquired the data under the supervision of Wei‐Guo Hu. De‐Wei An performed statistical analyses and together with Fei‐Ka Li and Ji‐Guang Wang prepared the first draft of the manuscript. Qian‐Hui Guo, Yi‐Qing Zhang and Jia‐Ye Qian contributed to the data processing. All authors critically commented and revised the manuscript and gave the final approval.
